# The association between maladaptive daydreaming and eating and obsessive-compulsive disorders in the general population: the mediating role of alexithymia

**DOI:** 10.1007/s00406-025-02083-z

**Published:** 2025-08-30

**Authors:** Alessia Renzi, Bleona Bytyqi, Rachele Mariani

**Affiliations:** Department of Dynamic and Clinical Psychology and Health Studies, “Sapienza” University of Rome, Via degli Apuli 1, 00185 Rome, Italy

**Keywords:** Maladaptive daydreaming; eating disorder, Obsessive-compulsive disorder, Alexithymia, Affect regulation, General population

## Abstract

Maladaptive daydreaming (MD) is a recently defined clinical condition characterized by an excessively immersive utilization of fantasy and imagination which can significantly impact both professional and social life. International literature has linked MD to obsessive-compulsive symptoms, but its association with alexithymia remains largely unexplored, and its connection to eating disorder symptoms is even more neglected. As a marker of affect regulation difficulties, alexithymia may heighten the risk and severity of MD, eating disorders, and obsessive-compulsive symptoms. Therefore, the general aim of the present study is to contribute to the study of MD in the general population, by examining potential associations between MD levels, eating disorders, obsessive-compulsive symptoms, and affect regulation capabilities. Additionally, the study aims to examine whether alexithymia mediates the relationship between eating and obsessive-compulsive symptoms, respectively, and MD levels. 562 participants (mean age = 27.16 years; SD = 10.21; 68% females) completed an online survey comprising: a socio-demographic questionnaire, the Maladaptive Daydreaming Scale (MDS), the 20-item Toronto Alexithymia Scale (TAS-20), the Eating Attitude Test (EAT-26), and the Obsessional Beliefs Questionnaire (OBQ-44). MDS score was positively related to TAS-20 total, difficulties in identifying and describing feelings as well as with eating disorders and obsessive-compulsive symptoms. Age showed a weak negative association with MDS. Regarding the mediation models, alexithymia significantly amplified the impact of eating/obsessive-compulsive symptoms on MD. The associations emerging by present study seem to support a common functioning of these clinical conditions based on maladaptive/compulsive behaviours/thought/fantasy, as strategy to regulate painful emotions. Further studies comparing these clinical populations are still needed.

**Supplementary Information:**

The online version contains supplementary material available at 10.1007/s00406-025-02083-z.

## Introduction

Maladaptive daydreaming (MD) is a clinical condition characterized by an excessively immersive use of fantasy and imagination, involving detailed and vivid scenarios that significantly impact an individual’s daily life [49, 56]. Daydreaming is a common way to manage emotions promoting relief and relaxation, allowing also the daydreamer to experience positive feelings [46]. Moreover, engaging in fantasizing and daydreaming can help individuals develop greater empathy and better understand emotions, as these skills may improve through the anticipation and imagination of social scenarios [1, 6, 8]. Nevertheless, some people can develop a compulsive dependence on it, dedicating an excessive amount of time each day to their inner fantasies. Indeed, individuals with MD spend numerous hours each day immersed in their fantasy worlds, experiencing intense sensations of presence and vividness while maintaining a dual awareness that keeps them connected to the external world [8, 52, 56]. They are aware of the distinction between reality and fantasy, as long as their creative imagination does not impair their judgment of what is real [52, 56]. However, the compulsive pattern of becoming deeply absorbed in detailed fantasies, often accompanied by repetitive movements, turns maladaptive when it begins to significantly disrupt daily functioning [52]. MD impacts professional and interpersonal relationships and other aspects of daily functioning [49, 63]. Preliminary findings from a population study estimate that approximately 2.5% of people experience clinical-level impairment due to these immersive daydreams, with a higher prevalence among young adults, peaking in the 18 to 30 age group [53].

This syndrome, linked to disorders like ADHD, anxiety, depression, and Obsessive-Compulsive Disorder (OCD) [57], was initially associated with dissociative and personality disorders [55]. Recently, it has been classified into four categories: dissociative disorder, attention disturbance, obsessive-compulsive spectrum disorder, and behavioral addiction [36, 54]. Maladaptive daydreamers show similarities with disorders characterized by excessive and compulsive fantasy and overthinking, similar to OCD and certain eating disorders. As precisely regards MD-OCD association, the high co-occurrence [56] and comorbidity rates (53.9%; [57] seem indicate shared features, such as obsessive preoccupation and repetitive behaviors [58]. Indeed, [45] found that checking and repetition compulsions are common between MD and obsessive-compulsive spectrum syndrome (OCSS). Moreover, a further common element between MD and OCSS is the presence of dissociative mechanisms, such as dissociative absorption, which lead to altered embodiment, intrusive thoughts, and impaired mental control when transitioning back to reality [45]. As specifically regards the association between EDs and MD there is a general lack of studies in the international literature; thus this topic needs investigations and actually only hypotheses are possible. However, some considerations can be done in the direction of linking all this clinical conditions together. These disorders might initially appear distinct, however a closer look reveals that the conditions feature repetitive thoughts and preoccupations about certain feared stimuli (such as food, body image, weight in EDs as well as symmetry, contamination, etc. in OCD, missing desirable experience in real life etc. in MD), typically followed by negative emotions [2]. In all these condition, the negative inner state faced lead to compensatory behaviors aimed at reducing anxiety or distress, such as immersive and dysfunctional fantasy (MD), restriction or binge/purge cycles for eating disorders (EDs) and handwashing or checking (OCD) [2]. In this light, an element linking OCD, EDs to MD is the difficulty in affect regulation, which may encourage the use of compulsions related to fantasy (MD), thinking (OCD), or eating habits (Eds) as a strategy to cope with and regulate negative emotional states, with specific characteristics for each condition.

In this direction, alexithymia, a term introduced by psychiatrist Peter Sifneos in the 1970 s, refers to challenges in identifying and expressing emotions and distinguishing between bodily sensations and emotions [41]. More recently it has been redefined as affect regulation disorder [59] and associated with traits such as low emotional awareness, an externally oriented thinking style, and reduced imaginative activities [38, 39]. In the international literature, it is recognized that a high alexithymia level is a vulnerability element for various mental and physical pathological conditions [3, 11, 27, 43].

Regarding its specific association with MD the literature is reduced and relatively recent whereas a wider part it focused on the association with the construct of affect regulation in a broader sense than alexithymia. Investigation on this topic demonstrated that maladaptive daydreaming can help individuals to regulate their emotions to some degree and create a buffer against both external and internal realities [63]. Preece and colleagues [38], focusing on variables like the frequency and vividness of daydreams, their content, and their role in emotion regulation attempts, found that individuals with high alexithymia tend to daydream more often, using their fantasies as a coping mechanism for emotional distress. In the same direction, [49] found a positive association between alexithymia and MD levels. In this light, Greene and colleagues [20] explored the connections between problematic daydreaming symptoms and difficulties in emotion regulation, finding that poorer emotion regulation abilities were associated with higher levels of maladaptive daydreaming symptoms. An intriguing result from their research was the negative correlation between lack of emotional clarity and the enjoyment of daydreaming. This suggests that the more enjoyable the daydreaming experience becomes more enjoyable, emotions may become more manageable, potentially leading to greater emotional clarity [20]. [13] found that, compared to non-MDers, MDers showed significantly higher scores in expressive suppression but not in cognitive reappraisal emotion regulation strategies.

As specifically regards the association between alexithymia and OCD and EDs the literature appears consistent. Alexithymia is believed to contribute to emotional challenges in individuals with eating disorders [10] and plays a role in both the onset and persistence of EDs [11, 61]. EDs are associated with struggles in emotional engagement and articulation showing a reduced capacity for imaginative thinking and difficulties in verbalizing their feelings [3]. A comprehensive literature review confirms that alexithymia is prevalent across eating disorders [32]. Furthermore, individuals with EDs generally exhibit higher levels of alexithymia compared to healthy controls [45, 64]. The high prevalence of emotional dysregulation reported by EDs patients may potentially result in a greater dependence on daydreaming as a dysfunctional regulatory mechanism [45].

Similar findings emerged about the association between alexithymia and OCD, with several studies showing high levels of alexithymia in this clinical condition [23, 42–44]. Other studies [7, 19] found that OCD patients had higher alexithymia levels than the healthy control. Meanwhile, Pozza and colleagues [37] discovered that overall alexithymia was linked to ordering and pure obsession symptoms, while difficulty identifying feelings (DIF) was associated with hoarding and checking symptoms showing that to different alexithymic dimensions correspond different OCD symptoms. In Tang and colleagues’ (2020) study, the main alexithymic features (difficulties in identifying and describing feelings, as well as externally orientated thinking) were all associated with obsessive thoughts, but not compulsive behavior. Moreover, Bagheri and colleagues [5] found that alexithymia had a significant positive correlation with OCD, as well as playing a role as a mediator of the relationship between coping styles and personality traits with OCD.

## Aims

In conclusion, research on MD has advanced significantly over the past decade. However, substantial gaps remain in the literature, limiting a comprehensive understanding of MDers and their functioning. Specifically, studies on the association between MD and OCD symptoms, as well as the role of alexithymia, are scarce, while research examining its link to EDs is entirely lacking. Consequently, there is a clear absence of studies comprehensively investigating these constructs or exploring the potential mediating role of alexithymia in the relationship between OCD, EDs, and MD. Considering these gaps, the present study aims to address them by examining these associations within a general population context. Specifically, this study investigates:

- The prevalence of MD (as defined by an MDS score above the clinical cut-off);
- Associations between MD levels and EDs and OCD symptomatology and alexithymic features;
- The role of alexithymia in mediating the association between EDs and OCD symptomatology and MD, respectively.

We hypothesize significant positive correlations among the investigated dimensions, with higher MD scores being associated with greater levels of OCD and ED symptoms, as well as higher alexithymia scores. Regarding the mediation models, we hypothesize that alexithymia will mediate the relationship between OCD and EDs symptoms, respectively, and MD. Considering that difficulties in affect regulation may increase reliance on maladaptive coping strategies when facing stressful conditions—such as heightened OCD and ED symptoms—this may, in turn, elevate the risk of developing or exacerbating various clinical conditions.

## Method

### Participant and procedure

The present investigation was conducted in the period between November 2023 and March 2024, and it was carried out in accordance with the code of ethics of the World Medical Association (Declaration of Helsinki) for experiments involving humans. Ethical approval was granted by the Ethics Committee of our University Department. To ensure consistent participation in the study, an online survey using a Type-form platform using the English language was realized. The survey was disseminated on several social media (Facebook, Instagram, etc.) and web pages, usually followed by young people since MD is prevalent among young adults. Participants gave their informed consent before completing the online survey. The inclusion criteria were:

- An age between 18 and 60 years old;
- Fluent in the English language;
- Giving the informed consent.

605 individuals completed the online survey, and forty-three responses (7.1%) were excluded due to duplication, distortion in the answers (where participants consistently followed a repetitive pattern as 1-2-3-4-5, repeated multiple times, or selected only one response option as all 1 s in one test, all 2 s in another, all 3 s in a third), or not meeting inclusion criteria (age not included in the range of interest). This resulted in a final sample size of 562 participants, with a mean age = 27.16 years (SD = 10.21) 68% females. Table 1 provides an overview of the sociodemographic characteristics of the group as well as Table  mean levels of the psychological dimensions investigated (see Table 1).

**Table 1:** Participants’ sociodemographic and psychological characteristics

Variables		*N*	(%)
Gender	Female	382	68.0
	Male	180	32.0
Nationality	Italian	200	35.7
	Other	362	64.3
Civil status	Married	59	10.6
	Single	412	73.2
	Divorced	16	2.8
	Cohabitant	75	13.4
Educational level	High School	165	29.5
	Student College	115	20.5
	Bachelors	180	32.1
	Master	86	15.2
	PhD and above	16	2.8
Employment status	Student	305	54.3
	Employed	191	34.0
	Freelance	36	6.3
	Not occupied	30	5.3

Drugs assumption	No	457	81.1
	Yes	105	18.9
		Mean	SD
MDS-16		29.91	18.52
TAS-20 total		50.71	11.67
EAT-26 total		10.64	10.77
OBQ-44 total		157.33	40.46

*MDS-16* Maladaptive Daydreaming Scale-16 item, *TAS*- *20* 20 item Toronto Alexithymia Scale, *EAT*-*26* Eating Attitude Test-26 item, *OBQ*-*44* Obsessive Belief Questionnaire-44 item

### Measures

**Socio-demographic questionnaire** was used to collect data on demographic variables such as age, gender, educational level, civil and working status, nationality and drug assumption.

**The 20-Item Toronto Alexithymia Scale (TAS-20)** [4, 9] is a self-administered questionnaire designed to measure alexithymia. Respondents rate each of the 20 items on a five-point Likert scale, from ‘strongly disagree’ (1) to ‘strongly agree’ (5). The TAS-20 provides an overall score and individual scores for three distinct factors: difficulty in identifying feelings (F1), difficulty in describing feelings (F2), and externally oriented thinking (F3). The total score can range from 20 to 100, with higher scores indicating greater alexithymia. The TAS-20 has demonstrated good internal consistency (Cronbach’s alpha for total score = 0.75) and test-retest reliability (*r* = 0.83). In the current study, a Cronbach’s alpha of 0.80 was observed for the total score.

**Obsessive Beliefs Questionnaire-44 (OBQ-44)** (Obsessive Compulsive Cognitions Working Group 33 is a self-assessment questionnaire consisting of 44 items designed to evaluate belief domains linked to OCD. Its revised version includes three subscales: responsibility/threat estimation (RT) with 16 items; importance/control of thoughts (ICT) with 16 items; and perfectionism/certainty (PC) with 12 items. Participants are asked to rate the extent to which they agree with each statement on a seven-point scale (1 = strongly disagree; 7 = strongly agree). The total score ranges from 44 to 308 with higher scores indicating a greater presence of obsessive beliefs. Studies have reported good psychometric properties in both clinical and non-clinical samples in different language versions (i.e. good validity, internal consistency, and test-retest reliability) [22, 30, 62]. In the current study, a Cronbach’s alpha of 0.94 was observed for the total score.

**The Eating Attitudes Test- 26 item** **(EAT-26) **[17] is a self-report questionnaire comprising 26 items, and three factors have been extracted from the previous version with 40 items (EAT 40): (a) dieting, (b) bulimia and food preoccupation and (c) oral control. Participants respond using a 6-point Likert scale, ranging from 1 = “never” to 6 = “always”. Several studies have examined the psychometric characteristics and have confirmed its high sensitivity to detect cases of EDs and its elevated reliability (Cronbach alpha = 0.80) of the questionnaire in subjects either with eating disorders or normal eating behavior [16, 17, 50]. In the current study, a Cronbach’s alpha of 0.88 was observed for the total score.

**The 16-item Maladaptive Daydreaming Scale** **(MDS-16)** [48, 56] is a self-report instrument consisting of 16 items designed to identify maladaptive daydreaming. Respondents are asked to answer on a scale ranging from 0 (never/none of the time) to 100% (extremely frequent/all the time), with 10% intervals. The questionnaire’s authors recommend a cut-off score of 40 to differentiate individuals with and without self-diagnosed maladaptive daydreaming, which is the widely recognized and used standard. The instrument demonstrates good psychometric properties [48, 56]. In the present study, the overall scale has a Cronbach’s alpha of 0.92.

### Statistical analysis

All statistical analyses were executed using the Statistical Package for Social Science version 25 for Windows (SPSS version 25; IBM, Armonk, NY, USA). Data were reported as frequency and percentage for discrete variables and as means and standard deviations for continuous variables. Pearson’s correlation analysis was performed to measure associations between psychological variables as well as age and gender. Mediation analyses were conducted to examine the direct effect of eating disorder and obsessive-compulsive disorder symptoms respectively on MD levels. Also, they examined the indirect effect of these symptoms on MD through affect regulation capabilities, as defined by the alexithymia construct. Age was inserted as a covariate. Mediation models were tested using SPSS macro, PROCESS. In particular, a series of linear regression models were fitted, and the size and significance of the indirect effects were estimated by a bootstrap procedure. A p-value < 0.05 was considered significant.

## Results

Data analysis showed that 29% of participants reported a score above the clinical cut-off for MD (total score ≥ 40).

As regards correlation analysis, several significant associations between MDS and psychopathological symptomatology as well as alexithymia levels emerged (See Table 2).

**Table 2:** Correlations between maladaptive daydreaming scale (MDS–16) scores and psychological and demographic variable

	MDS-16
Age	− 0.183*
Gender	− 0.004
TAS-20	0.337*
DIF	0.296*
DDS	0.406*
EOT	0.058
EAT-26	0.235*
Dieting	0.163*
Bulimia	0.268*
Oral control	0.184*
OBQ-44	0.249*
Perfectionism/certainty (PC)	0.217*
Importance/control of thoughts (ICT)	0.210*
Responsibility/threat estimation (RT)	0.220*

*TAS-20* 20-item Toronto Alexithymia Scale, DIF Difficulty in Identifying Feelings, DDF Difficulty in Describing Feelings, EOT Externally Oriented Thinking *EAT-26*  Eating Attitude Test-26 item, Obsessive Beliefs Questionnaire-44; Maladaptive Daydreaming Scale-16 item

*p < 0.01

**Fig. 1:**
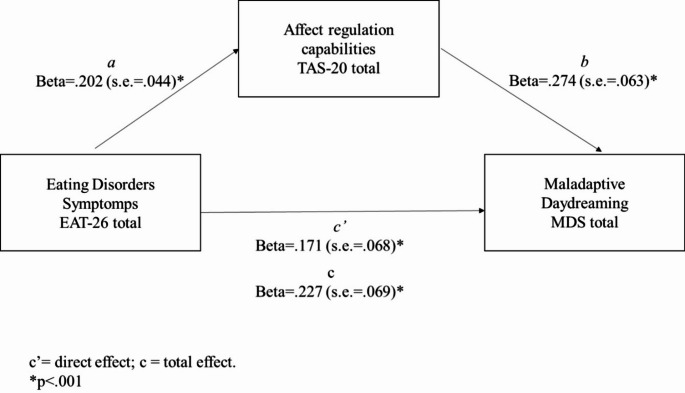
Mediation model for testing direct and indirect effect of eating symptoms on MDS

Model 4 of the PROCESS macro [21] was used to investigate whether the indirect effect of alexithymia on the link between EDs symptoms (EAT-26 total) and MD (MDS) was significant (see Fig. 1). Age was considered a covariate for the prediction of both the MDS and the TAS-20 total scores. Results showed a significant total effect of EDs symptomatology on MDD levels (b = 0.388; se = 0.069; Beta = 0.227; *p* < 0.0001). Moreover, results showed a significant direct effect of eating disorders symptomatology (EAT-26 total) on alexithymia (TAS-20) total score (b = 0.219; se = 0.044; Beta = 0.202; *p* < 0.0001) and of age (b = − 0.156; se = 0.046; Beta = − 0.137; *p* < 0.001). The direct effect of the alexithymia total levels on the MDS scores was also significant (b = 0.435; se = 0.063; Beta = 0.275; *p* < 0.0001) as well as the direct effect of the EDs symptomatology on the MDS level (b = 0.298; se = 0.069; Beta = 0.173 *p* < 0.0001), with a significant value emerging for age (b = − 0.254, se = 0.070, Beta = − 0.140; *p* < 0.001). Finally, the indirect effect was significant (b = 0.095, completely standardized indirect effect: Beta = 055; bootstrap s.e. = 0.024, bootstrap 95% C.I.: 0.0298–0.0854). Therefore, eating disorders symptomatology demonstrated also an indirect effect on maladaptive daydreaming levels through affect regulation capabilities.

The overall model indicates that the predictors accounted for 16% (R2 = 0.157) of the variance observed in MDS scores (F_(3, 556)_ = 34.656; *p* < 0.0001).

**Fig. 2:**
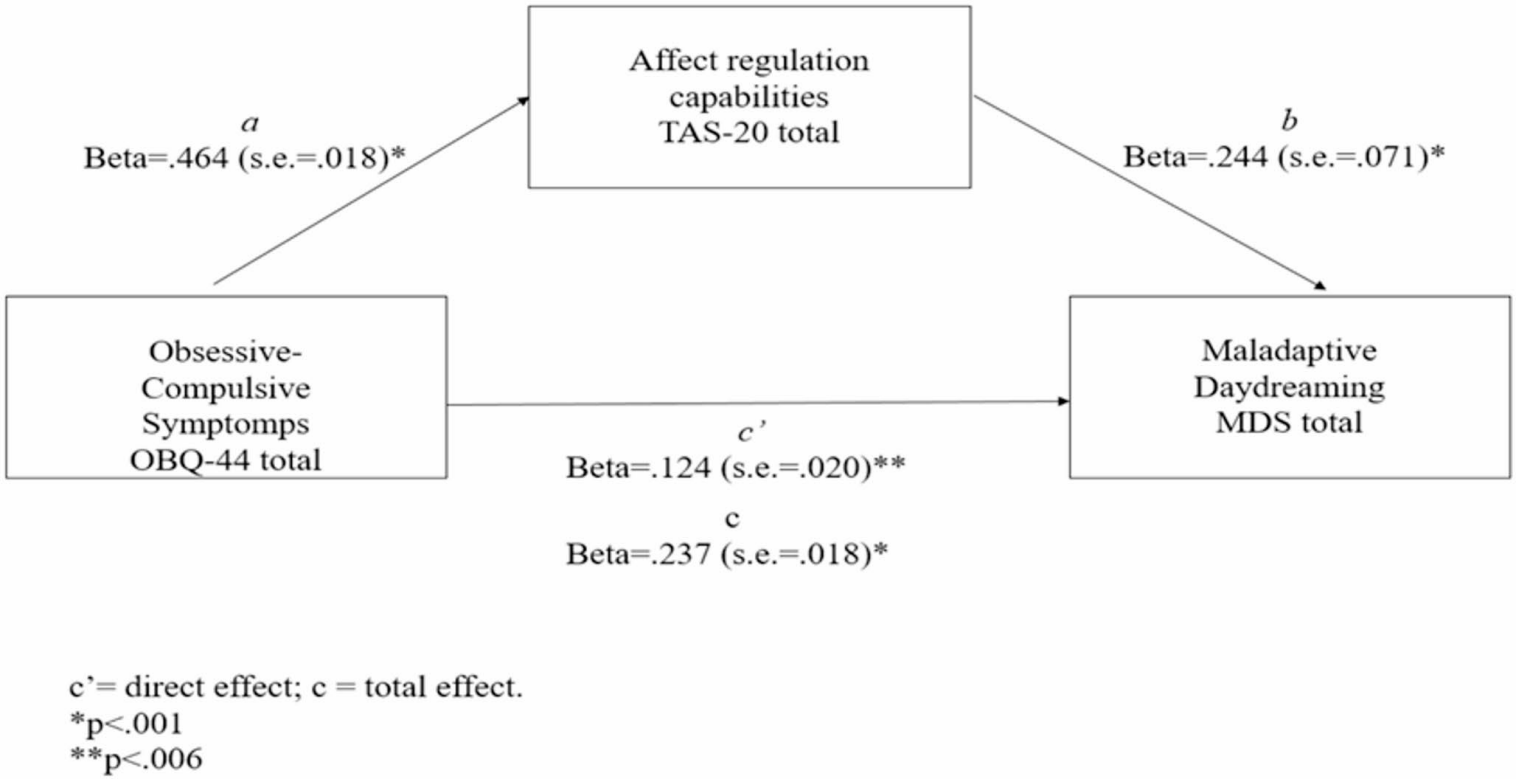
Mediation model for testing direct and indirect effect of obsessive-compulsive symptomps on MD

A further model was tested to investigate whether the indirect effect of alexithymia on the link between OCD symptoms (OBQ-44 total) and MDD (MDS) was significant (see Fig. 2). Age was considered a covariate for the prediction of both the MDS and the TAS-20 total scores. Results showed a significant total effect of obsessive-compulsive symptomatology on MD levels (b = 0.107; se = 0.018; Beta = 0.237; *p* < 0.0001). Moreover, results showed a significant direct effect of obsessive-compulsive symptomatology (OBQ-44 total) on alexithymia (TAS-20) total score (b = 0.133; se = 0.010; Beta = 0.464; *p* < 0.0001) and of age (b = − 0.097; se = 0.042; Beta = − 0.086; *p* = 0.02). The direct effect of the alexithymia total levels on the MDS scores was also significant (b = 0.386; se = 0.071; Beta = 0.244; *p* < 0.0001) as well as the direct effect of the obsessive-compulsive symptomatology on the MDS level (b = 0.056; se = 0.020; Beta = 0.124 *p* < 0.006), with a significant value emerging for age (b = − 0.238, se = 0.071, Beta = − 0.133; *p* < 0.001). Finally, the indirect effect was significant (b = 0.051, completely standardized indirect effect: Beta = 0.113; bootstrap s.e. = 0.010, bootstrap 95% C.I.: 0.0316–0.0713). Therefore, obsessive-compulsive symptomatology also demonstrated an indirect effect on maladaptive daydreaming levels through affect regulation capabilities. The overall model indicates that the predictors accounted for 13% (R2 = 0.134) of the variance observed in MDS scores (F_(3, 554)_ = 28.709; *p* < 0.0001).

## Discussion

Despite progress in MD research in the last decade, key gaps remain, including limited studies on its link to OCD, alexithymia, and the absence of research on its association with EDs. Moreover, no studies have jointly examined these constructs or alexithymia’s mediating role. Consistent with existing literature, our findings reveal a strong link between youth and MD symptoms [53]. Recent studies suggest a link between prior psychopathological conditions and the function of maladaptive daydreaming [29, 55]. Our first two hypotheses were to test the relationships between MDs and EDs, in parallel between OCD and MD. The first relevant finding that emerged is how the daydreaming symptom correlates with both disorders. From our first result, we could argue that MD is a symptom closely related to the two disorders. Therefore, we propose that MD may serve as a particularly sensitive indicator of a broader and more complex psychopathological profile. In this context, MD reflects not only a tendency toward immersive and highly elaborate internal fantasy activity, but also a marked withdrawal from external reality—both of which may signal underlying emotional dysregulation or comorbid psychological conditions. Indeed, we simultaneously explore the connection between alexithymia and MD. Alexithymia, as an emotional dysregulation problem, could be seen as a base for emotional difficulties under the serval psychopathology spectrum. The literature has well highlighted the relationship between EDs and OCD and alexithymia [5, 26]. Additionally, MD has been poorly studied in relation to alexithymia, nevertheless some studies have highlighted that MDers have an underlying difficulty in managing, regulating, and naming internal emotional experiences, especially when these emotions are dysregulated and negative [12, 45, 57]. In our results, we found a positive relation between MD and alexithymia, as well as MD versus OCD and EDs. A noteworthy finding regarding the relationship between alexithymia and MD is the significant positive correlation with all factors, except for the EOT factor, where no significant correlation was observed. This finding aligns closely with the one emerging from [39] who suggest that the Externally Oriented Thinking (EOT) factor is largely unrelated to the capacity for immersive daydreaming. Their results underscore the distinction between different cognitive styles involved in emotional dysregulation. Specifically, MD appears to rely more heavily on the other two alexithymia dimensions—Difficulty Identifying Feelings (DIF) and Difficulty Describing Feelings (DDF)—which are more closely tied to internal emotional processing. This suggests that MD may be more associated with inward-focused, imaginative cognitive activity rather than externally directed thinking patterns. Our findings allowed us to explore two models (Figs. 1 and 2) to further investigate alexithymia’s role in MD. We tested the direct effects of EDs and OCD on MD, as well as the indirect effects mediated by alexithymia. To delve deeper into this aspect, we performed two mediation models using alexithymia as a connecting construct between these symptoms. Our significant models show that there is a direct relationship between EDs and OCD, stronger in OCD, but there is also a relationship between the two explained by the inability to name affects, i.e., alexithymia. More precisely, the evaluation of the standardized coefficients (direct, indirect, and total) allows us to conclude that alexithymia amplifies the effects of both ED and OCD symptomatology on MD scores. This may support findings from international literature highlighting that deficits in the emotional regulation capabilities represent an increased risk factor for both the development and the exacerbation of clinical conditions [25].

In the same direction as our results, several researchers highlight the relationship between MD and aspects of personality traits such as borderline or narcissistic aspects that are related to MD [18, 35]. These results emphasize that the maladaptive functioning of daydreams seems to be linked to difficulties in emotional management. Psychodynamic theories further suggest that these difficulties are closely related to the regulation of internal conflicts. Our results emphasize that psychopathological functioning like EDs and OCD, which involve compulsive strategies, are closely associated with the compulsive production of fantasies. This connection may be rooted in an underlying element of emotional dysregulation that is common across various psychopathological disorders. Thus, it seems that a common factor underlying daydreaming is precisely difficulty in regulating emotions and being in touch with them. This result is consistent with the literature that highlights how emotional dysregulation is a factor that cuts across many psychopathologies where the compulsive and dependent dimension is central.

Regarding the rate of participants reporting an MDS score above the clinical cut-off (29%), some considerations can be made by comparing this data with findings from the international literature. In a general population samples from Israel, prevalence ranges from 5.5 to 8.5% in younger age groups [51], whereas in clinical populations, the percentage is significantly higher. For instance, among individuals diagnosed with attention deficit hyperactivity disorder (ADHD), nearly 46% score above the clinical threshold [60]. Additionally, studies recruiting participants from online communities specifically dedicated to MDers have reported even higher prevalence rates, reaching up to 70% [20]. This highlight the need for further studies exploring MD in general population. Regarding the other constructs investigated, comparing the mean scores obtained in the present study with those reported in previous international research requires specific consideration for each condition. Qualitatively, the sample’s mean OBQ-44 score (M = 157.33) appears slightly lower than those reported in clinical OCD populations across different countries (e.g., [22] M = 176.52; [28] M = 178.26; [62] M = 178.23), but notably higher than scores from non-clinical populations [22] M = 106.54; [62] M = 107.19). A similar trend is observed for alexithymia scores (M = 50.71), which align closely with those reported in various clinical samples involving both mental and physical pathological conditions [9] M = 53.62; [24] M = 57.47; [31] M = 50.43). On the contrary the obtained mean scores on EAT-26 (M = 10.64) align with those emerging from study on general population [34] M = 9.37; [14] M = 11.3).

Given all these elements discussed in our work many clinical implications can be addressed. Our results together with other studies cited above highlight how MD can be a trans-diagnostic clinical indicator also linked to transversal functioning such as Alexithymia and emotional regulation as well as character traits that identify deeper personality mechanisms [40]. In other words, MD symptoms could be used as early markers of complex psychopathologies, reflecting a common pattern of withdrawal from reality and emotional dysregulation. Furthermore, given the close relationship between ED and ODC from a clinical perspective, it would be useful to explore the content of daydreaming fantasies in association with these psychopathologies in order to understand the underlying emotional regulation functioning. Furthermore, as alexithymia mediates the relationship between MD, EDs and OCD. In particular, difficulties in identifying (DIF) and describing (DDF) emotions play a key role, this result implies an important clinical indication, namely that psychotherapeutic interventions (e.g. emotion-focused therapy, mindfulness, psychodynamic therapy or DBT) should work on alexithymia to reduce dysfunctional daydreaming. In this way, clinicians could understand whether excessive daydreaming has an escape function from disturbing emotions, and thus be able to intervene on the origin of the emotional distress rather than only on the symptom itself. This result also leads to exploring future directions as MD shares compulsive aspects with disorders such as OCD and certain types of ED (e.g. binge eating), suggesting a similar mechanism of mental addiction or compulsion.

### Limitations

While this study offers several novel insights into MD, there are some limitations to consider: (a) The “snowball” recruitment technique used on institutional websites and social media may introduce selection bias, particularly in reaching individuals who are less familiar with technology, lack internet access, or do not use certain social media platforms; (b) reliance on self-report questionnaires could lead to response bias if participants misrepresent their answers; (c) the use of English, which may not be the native language for many participants, could have introduced some bias related to the accurate understanding of the items; and (d) the cross-sectional design, meaning conclusions should be interpreted cautiously. Although mediational analyses provide a deeper understanding of the relationships between variables, they do not establish causality. Future longitudinal studies are needed to confirm and further explore these findings, as the evidence presented here is preliminary.

## Conclusion

Our correlational and cross-sectional study reveals a direct relationship between eating disorders, OCD, and maladaptive daydreaming (MD), particularly in younger populations. More significantly, we found that beyond these direct links, MD is also influenced by emotional dysregulation, such as alexithymia. This connection suggests that difficulties in processing emotional information contribute to the compulsion to fantasize. These issues may arise from traumatic experiences, dissociation, or internal conflicts that trigger defense mechanisms or emotional disconnection, common across various psychopathological conditions. In summary, our findings indicate that maladaptive fantasy is closely associated with other psychopathological disorders. In conclusion, our sample suggests that maladaptive fantasy is closely linked to other psychopathological disorders. Additionally, this phenomenon seems to arise from challenges in emotional processing, which may intersect with various psychological, personality, and severe psychopathological conditions. This is consistent with psychoanalytic theory and empirical research, which suggest that trauma survivors often struggle to adapt to reality, leading to the development of specific conscious and unconscious maladaptive mechanisms to cope with traumatic and conflicted memories, as well as the associated painful emotions [15]. In particular, this highlights a vicious cycle where conflicting and/or traumatic memories remain insufficiently processed, leading to dysregulated emotional activations that continuously require management [49].

Present study aims at overcoming some key gaps in the international literature regarding the investigation of the selected conditions and constructs in association with MD with a focus on alexithymia’s mediating role. This appears relevant since it is a first contribution offering a comprehensive evaluation aof these elements trying helping clinicians and professionals in understanding if maladaptive daydreaming is a distinct syndrome or a maladaptive symptom present in various other psychopathologies. Present findings confirm the strong association of MD with other clinical and emotional regulation capabilities, nevertheless the mediation models tested explained a percentage of 13–15% of variance in MD score and while this suggests a moderate effect, it also implies that other unexamined factors contribute to MD, highlighting the need for further research to identify additional influences.

The clinical implications of alexithymia’s mediating role are significant. Implementing broad clinical interventions aimed at improving emotional regulation and coping with negative affects, especially in young adults, could help prevent the development of compulsive behaviors [47].

## Supplementary Information

Below is the link to the electronic supplementary material.

Supplementary Material 1

## Data Availability

The data supporting the findings of this study are available upon reasonable request from the corresponding author [AR].

## Abbreviations

MD: Maladaptive daydreaming
MDers: Maladaptive daydreamers
DIF: Difficulty identifying feelings
MDS: Maladaptive Daydreaming Scale
EDs: Eating disorders
EAT-26: Eating attitude test
OCD: Obsessive compulsive disorder
OBQ-44: Obsessional Beliefs Questionnaire
RT: Responsibility/threat estimation
ICT: Importance/control of thoughts
PC: Perfectionism/certainty
TAS-20: Toronto Alexithymia Scale
F1: Difficulty in identifying feelings
F2: Difficulty in describing feelings
F3: Externally oriented thinking
