# Supplementary material for: The association between maladaptive daydreaming and eating and obsessive-compulsive disorders in the general population: the mediating role of alexithymia
Source: Eur Arch Psychiatry Clin Neurosci. 2025 Aug 30;276(4):1399–409. doi: 10.1007/s00406-025-02083-z (PMC13233910; doi:10.1007/s00406-025-02083-z)
Supplement: Supplementary file 1 — Supplementary Material 1 [file 406_2025_2083_MOESM1_ESM.docx]

**The association between Maladaptive Daydreaming and Eating and Obsessive-Compulsive Disorders in the general population: the mediating role of Alexithymia**

***Running title:*** *Maladaptive Daydreaming and affect regulation*

*Renzi Alessia* ^(a)^*, Bytyqi Bleona* ^(a)^*, Mariani Rachele* ^(a)^

^(a)^ Department of Dynamic and Clinical Psychology and Health Studies, “Sapienza” University of Rome, Via degli Apuli 1, 00185 Rome, Italy

**Correspondig Author:**

Alessia Renzi, Department of Dynamic and Clinical Psychology and Health Studies, “Sapienza” University of Rome

Via degli Apuli 1, 00185, Rome, Italy

Phone number: +39 0649917989

E-mail: [alessia.renzi@uniroma1.it](mailto:alessia.renzi@uniroma1.it)

ORCID: 0000-0002-8553-4444

**Declaration of competing interest:** none.

**Conflict of interest:** There are no conflicts of interests.

**Funding:** This research did not receive any specific grant from funding agencies in the public, commercial or not-for-profit sectors.

**Ethical approval:** The present study was conducted in accordance with the Declaration of Helsinki and approved by the Institutional Ethics Committee of Department of Dynamic and Clinical Psychology and Health Studies (Prot. n. 0001210 del 15/12/2020).

**Informed Consent Statement:** Informed consent was obtained from all subjects involved in the study.

**Data availability statement:** The data supporting the findings of this study are available upon reasonable request from the corresponding author [AR].
